# Genetic factors affect the susceptibility to bacterial infections in diabetes

**DOI:** 10.1038/s41598-021-88273-w

**Published:** 2021-05-04

**Authors:** Johan R. Simonsen, Annemari Käräjämäki, Anni A. Antikainen, Iiro Toppila, Emma Ahlqvist, Rashmi Prasad, Dina Mansour-Aly, Valma Harjutsalo, Asko Järvinen, Tiinamaija Tuomi, Leif Groop, Carol Forsblom, Per-Henrik Groop, Niina Sandholm, Markku Lehto

**Affiliations:** 1 Folkhälsan Institute of Genetics, Folkhälsan Research Center, Biomedicum Helsinki, Helsinki, Finland; 2 Abdominal Center, Nephrology, University of Helsinki and Helsinki University Hospital, Helsinki, Finland; 3 Research Program for Clinical and Molecular Metabolism, Faculty of Medicine, University of Helsinki, Helsinki, Finland; 4 Department of Primary Health Care, Vaasa Central Hospital, Vaasa, Finland; 5 Diabetes Center, Vaasa Health Care Center, Vaasa, Finland; 6 Department of Clinical Sciences, Lund University Diabetes Centre, Lund University and Skåne University Hospital, Malmö, Sweden; 7 National Institute for Health and Welfare, Helsinki, Finland; 8 Division of Infectious Diseases, Inflammation Centre, Department of Medicine, Helsinki University Hospital, Helsinki, Finland; 9 Abdominal Center, Endocrinology, University of Helsinki and Helsinki University Hospital, Helsinki, Finland; 10 Institute for Molecular Medicine Finland, Helsinki, Finland; 11 Department of Diabetes, Central Clinical School, Monash University, Melbourne, VIC Australia

**Keywords:** Infectious diseases, Genetic association study

## Abstract

Diabetes increases the risk of bacterial infections. We investigated whether common genetic variants associate with infection susceptibility in Finnish diabetic individuals. We performed genome-wide association studies and pathway analysis for bacterial infection frequency in Finnish adult diabetic individuals (FinnDiane Study; N = 5092, Diabetes Registry Vaasa; N = 4247) using national register data on antibiotic prescription purchases. Replication analyses were performed in a Swedish diabetic population (ANDIS; N = 9602) and in a Finnish non-diabetic population (FinnGen; N = 159,166). Genome-wide data indicated moderate but significant narrow-sense heritability for infection susceptibility (h^2^ = 16%, P = 0.02). Variants on chromosome 2 were associated with reduced infection susceptibility (rs62192851, P = 2.23 × 10^–7^). Homozygotic carriers of the rs62192851 effect allele (N = 44) had a 37% lower median annual antibiotic purchase rate, compared to homozygotic carriers of the reference allele (N = 4231): 0.38 [IQR 0.22–0.90] and 0.60 [0.30–1.20] respectively, P = 0.01). Variants rs6727834 and rs10188087, in linkage disequilibrium with rs62192851, replicated in the FinnGen-cohort (P < 0.05), but no variants replicated in the ANDIS-cohort. Pathway analysis suggested the *IRAK1* mediated NF-κB activation through IKK complex recruitment-pathway to be a mediator of the phenotype. Common genetic variants on chromosome 2 may associate with reduced risk of bacterial infections in Finnish individuals with diabetes.

## Introduction

Infectious diseases have had a great impact on mortality throughout human evolutionary history. Genetic traits offering protection from or susceptibility to infections have heavily affected the survivability and genetic selectivity of populations^1,2^. Despite modern medical technologies, including vaccines and antimicrobial therapies, infections still have a massive effect on global morbidity and mortality. Based on the recent World Health Organization (WHO) report, infections are the fourth most common cause of death globally and the most common cause of death in low-income countries^3^.

Previous research has demonstrated how genome-wide association studies (GWAS) can effectively be implemented to uncover genetic factors that increase susceptibility to infections^4–6^. However, GWAS reports on susceptibility to infections, have none the less been scarce^7^ and published studies in this field have mainly focused on certain specific infections or pathogens, such as HIV^8^, malaria^9,10^, hepatitis^11,12^ and tuberculosis^10,13,14^. Perhaps the most comprehensive study performed on susceptibility to infections using GWAS, found several loci located in the HLA-region on chromosome 6 to be associated with the susceptibility to common infections such as the common cold, pneumonia, streptococcal pharyngitis and urinary tract infections, in individuals with European ancestry^15^. Furthermore, GWAS have also demonstrated how genetic factors can play an opposite, protective role, in the immunity against infectious agents^16^.

Diabetes is a common disease with an increasing prevalence, at present affecting nearly 500 million people globally. Of note, several studies have demonstrated how diabetes increases the risk of infections^17–19^. Such increased susceptibility to infections may be mediated through defects in host immune defense mechanisms, including impaired neutrophil function^20,21^, which plays an essential role in the defense especially against bacterial pathogens. Indeed, we have previously found that individuals with type 1 diabetes are more susceptible to bacterial infections, and that individuals with type 1 diabetes have a roughly two times higher risk of contracting a bacterial infection compared to individuals without diabetes^22^. We also found that the risk of infections correlated with the stage of diabetic nephropathy, as well as with the glycemic control. Even though the increased risk of infections observed in diabetes is widely acknowledged, the underlying pathophysiological mechanisms are still largely unknown.

We hypothesized that a part of the increased susceptibility to bacterial infections in diabetes could be modulated by common genetic factors. In order to identify such factors, we utilized GWAS and national register data on antibiotic purchases from two separate, comprehensive Finnish diabetes study cohorts: The Finnish Diabetic Nephropathy Study (FinnDiane, type 1 diabetes) and the Vaasa Diabetes Registry (DIREVA, all types of diabetes).

## Results

**Figure 1:**
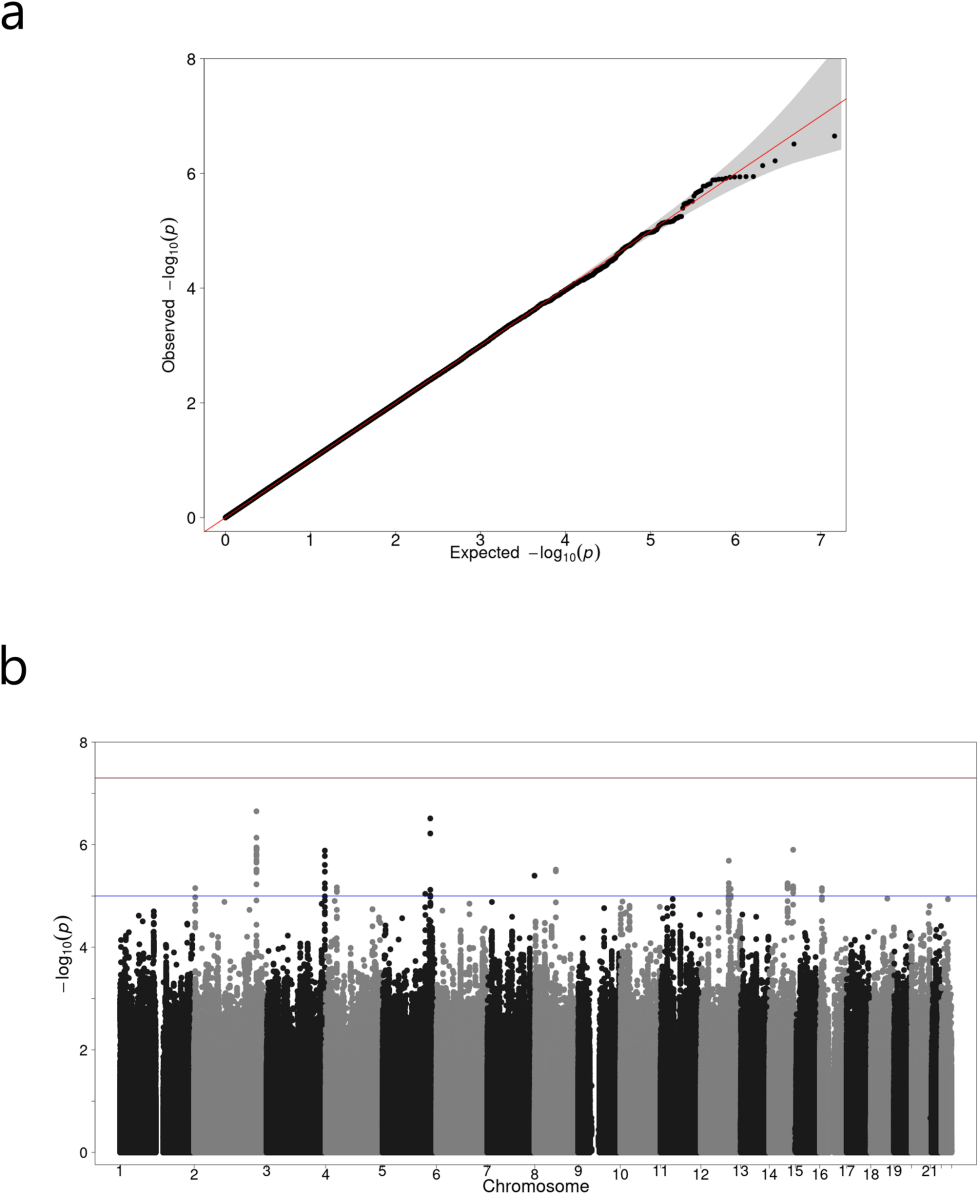
(**a**) Quantile–quantile and (**b**) Manhattan plots of the meta-analysis results of the GWAS in the FinnDiane-cohort and DIREVA-cohort. In (**b**) two horizontal lines indicate P value thresholds for suggestive significance (P < 10^–5^) and genome-wide significance (P < 5 × 10^–8^).

**Figure 2:**
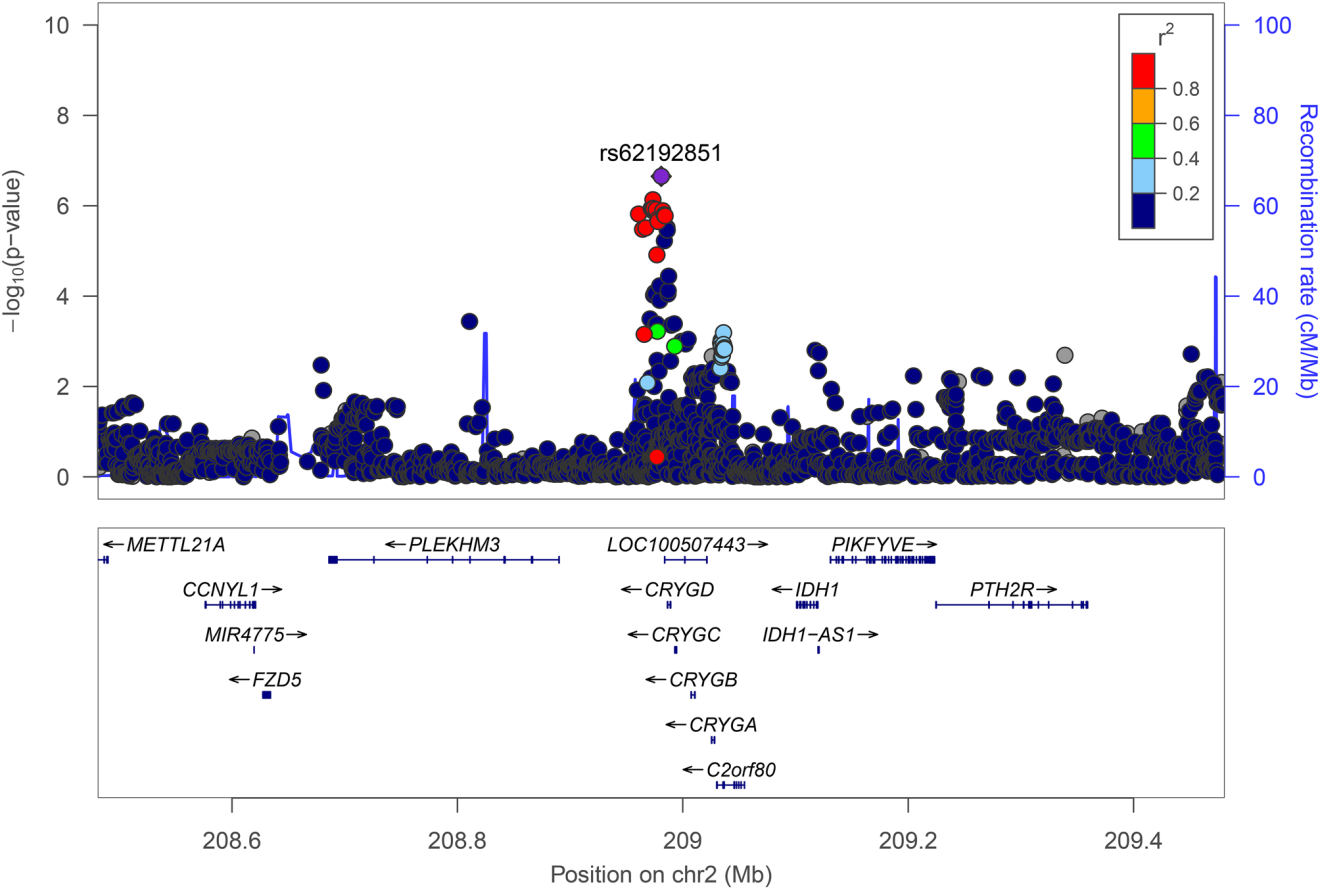
Regional locus zoom association plot of the associated region at rs62192851. The blue line denotes the recombination rate.

The median number of antibiotic purchases per follow-up year per subject was 0.8 (IQR 0.3–1.2) in the FinnDiane cohort and 0 (0–0.05) in the DIREVA-cohort, without logarithmic transformation (Table 1). Based on the FinnDiane cohort, the narrow sense heritability of the infection risk related phenotype adjusted for long-term glycemic control was 16.0% (SE: 0.08, P = 0.02). The quantile–quantile plots in all three analyses showed good adherence to the diagonal line of expected significance, and little excess genomic inflation was observed (Fig. 1a, Supplementary Fig S1). The meta-analysis yielded no GWAS significant loci, although numerous suggestive associations (P < 1 × 10^–5^) were discovered (Fig. 1b). The top single nucleotide polymorphism (SNP) (rs62192851) obtained a P-value of 2.23 × 10^–7^ in the meta-analysis (Table 2) and a similar effect size and level of significance in the FinnDiane and DIREVA-cohorts (β: − 0.13 [95% CI − 0.20 to − 0.07], P = 1.30 × 10^–4^ and − 0.12 [− 0.20 to − 0.05], P = 2.42 × 10^–3^, respectively). rs62192851 is located in a non-coding region, although in close proximity (< 100 kB) to several genes (Fig. 2). Within the lead locus, 20 other variants reached suggestive significance (P < 1 × 10^–5^) and were all in high linkage disequilibrium with the lead variant, likely reflecting the same association signal. In addition, 12 other loci reached suggestive P-values of < 1 × 10^–5^ (Table 2).

**Table 1:** Clinical characteristics of the individuals in the FinnDiane and DIREVA-cohorts.

Baseline demographics of the cohorts		
Variable	FinnDiane	DIREVA
N	5092	4247
Sex, n (% female)	2488 (48.8)	1868 (44.0)
Average age during follow-up (years)	42.3 (33.0–51.2)	63.9 (54.6–71.4)
Average HbA_1c_ during follow-up (mmol/mol)	68 ± 15	52 ± 13
Age at onset of diabetes (Years)	14.9 (9.4–24.3)	57.0 (46.0–64.8)
Systolic blood pressure (mmHg)	131 (121–143)	140 (130–150)
Diastolic blood pressure (mmHg)	80 (72–86)	80 (74–87)
eGFR (mL/min/1.73 m^2^)	102 (84–115)	81.5 (63.7–94.6)
LDL-cholesterol (mmol/L)	2.9 (2.4–3.5)	3.1 (2.5–3.8)
Subjects treated with exogenous insulin, n (%)	5092 (100)	1726 (40.6)
Proportion of T1D (%)	5092 (100)	545 (12.8)
Mean number of antibiotic purchases per year per subject	0.8 (0.3–1.2)	0 (0–0.05)
Subjects with < 1 antibiotic purchase in average per follow-up year, n (%)	3499 (68.7)	4074 (96.0)
Subjects with 1–3 antibiotic purchases in average per follow-up year, n (%)	1378 (27.1)	140 (3.3)
Subjects with > 3 antibiotic purchases in average per follow-up year, n (%)	215 (4.2)	24 (0.7)
Follow-up (years)	20 (19–20)	11.6 (7.1–18.3)

Data is presented as means ± SD, median (IQR) or number (percentages) where appropriate. In the DIREVA-cohort, data on blood pressure was available for 3231 individuals. *HbA*_*1c*_ glycated hemoglobin, *eGFR* estimated glomerular filtration rate, *LDL* low-density lipoprotein, *T1D* type 1 diabetes.

**Table 2:** The top variant of each locus reaching suggestive significance (P < 1 × 10^–5^) in the combined meta-analysis performed on the FinnDiane and DIREVA GWAS, as well as their results in the separate GWAS.

SNP	Chromosome:position	EA/OA	Meta-analysis			FinnDiane			DIREVA		
			EAF	β (95% CI)	P	EAF	β (95% CI)	P	EAF	β (95% CI)	P
rs62192851	2:208,980,851	A/G	0.09	− 0.13 (− 0.18 to − 0.08)	2.23 × 10^–7^	0.09	− 0.13 (− 0.20 to − 0.07)	1.30 × 10^–4^	0.09	− 0.12 (− 0.20 to − 0.05)	2.42 × 10^–3^
rs35451602	2:3,631,189	T/TAA	0.20	− 0.09 (− 0.12 to − 0.05)	7.03 × 10^–6^	0.20	− 0.08 (− 0.13 to − 0.03)	1.32 × 10^–3^	0.21	− 0.09 (− 0.15 to − 0.03)	3.13 × 10^–3^
rs36002025	3:195,278,355	T/C	0.43	− 0.08 (− 0.11 to − 0.05)	1.30 × 10^–6^	0.43	− 0.07 (− 0.11 to − 0.02)	2.29 × 10^–3^	0.44	− 0.10 (− 0.15 to − 0.05)	7.92 × 10^–5^
rs4099475	4:37,773,400	C/G	0.05	− 0.16 (− 0.23 to − 0.09)	6.79 × 10^–6^	0.05	− 0.15 (− 0.25 to − 0.05)	2.86 × 10^–3^	0.05	− 0.17 (− 0.27 to − 0.06)	2.30 × 10^–3^
rs141002783	5:143,269,636	C/G	0.02	0.27 (0.15 to 0.39)	9.09 × 10^–6^	0.02	0.19 (0.03 to 0.34)	1.50 × 10^–2^	0.01	0.36 (0.16 to 0.55)	3.03 × 10^–4^
rs4559039	5:160,233,534	A/C	0.03	− 0.26 (− 0.36 to − 0.16)	3.08 × 10^–7^	0.03	− 0.20 (− 0.34 to − 0.06)	5.73 × 10^–3^	0.03	− 0.29 (− 0.43 to − 0.15)	7.61 × 10^–5^
rs183041036	7:158,289,622	G/A	0.22	0.11 (0.06 to 0.15)	4.03 × 10^–6^	0.22	− 0.11 (− 0.15 to − 0.06)	1.38 × 10^–5^	0.04	− 0.12 (− 0.25 to − 0.02)	9.58 × 10^–2^
rs7833059	8:70,952,739	C/G	0.09	− 0.12 (− 0.17 to − 0.07)	3.06 × 10^–6^	0.09	− 0.14 (− 0.21 to − 0.06)	2.59 × 10^–4^	0.11	− 0.11 (− 0.19 to − 0.04)	2.82 × 10^–3^
rs11110058	12:100,210,357	A/G	0.19	− 0.09 (− 0.13 to − 0.05)	7.30 × 10^–6^	0.19	− 0.08 (− 0.13 to − 0.02)	4.68 × 10^–3^	0.18	− 0.12 (− 0.19 to − 0.06)	2.24 × 10^–4^
rs7316104	12:93,918,909	C/T	0.44	− 0.07 (− 0.1 to − 0.04)	2.05 × 10^–6^	0.44	− 0.07 (− 0.1 to − 0.02)	2.01 × 10^–3^	0.43	− 0.06 (− 0.11 to − 0.02)	7.03 × 10^–3^
rs11850643	14:100,193,684	A/G	0.03	− 0.27 (− 0.38 to − 0.16)	1.26 × 10^–6^	0.03	− 0.31 (− 0.45 to − 0.18)	5.03 × 10^–6^	0.01	− 0.18 (− 0.38 to 0.02)	8.22 × 10^–2^
rs2888050	14:81,588,015	T/G	0.20	0.08 (0.05 to 0.12)	5.65 × 10^–6^	0.20	0.08 (0.03 to 0.13)	2.24 × 10^–3^	0.19	0.09 (0.03 to 0.15)	2.28 × 10^–3^
rs1382484	16:7,218,129	G/A	0.08	0.12 (0.07 to 0.17)	7.04 × 10^–6^	0.08	0.13 (0.06 to 0.20)	3.99 × 10^–4^	0.09	0.09 (0.01 to 0.18)	2.67 × 10^–2^

*SNP* single nucleotide polymorphism, *EA/OA* effect allele/other allele, *EAF* effect allele frequency.

### The effect of the lead locus on infection susceptibility

**Figure 3:**
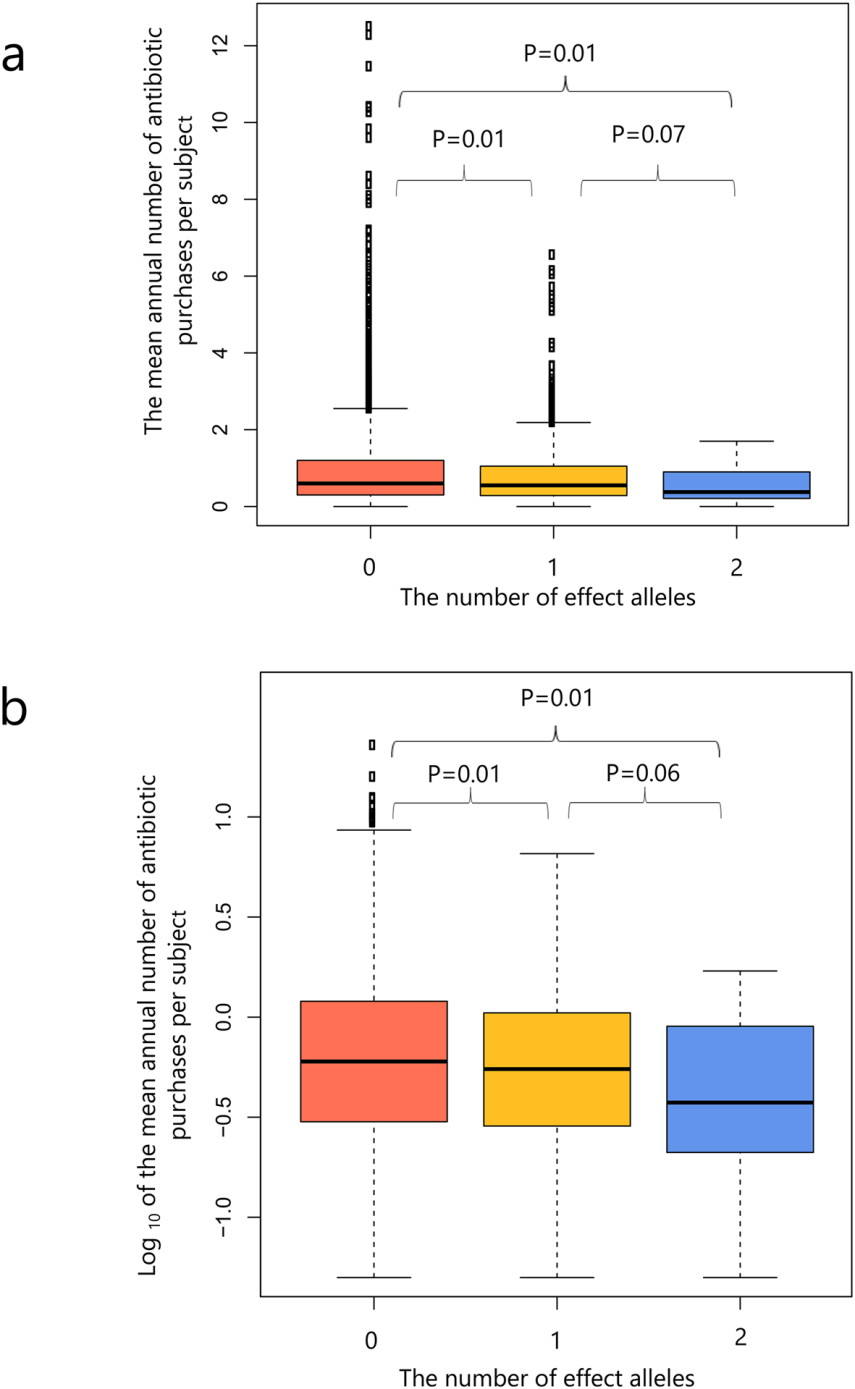
Boxplots demonstrating the median and interquartile range of (**a**) the mean annual number of antibiotic purchases per subject, and (**b**) the logarithmically transformed mean annual number of antibiotic purchases per subject, in individuals with 0 effect alleles (N = 4231), 1 effect allele (N = 817), and 2 effect alleles (N = 44) of rs62192851. Only individuals from the FinnDiane study were included. Differences between groups were investigated with the Mann–Whitney *U* test.

Interestingly, we observed a negative effect size for the top SNP, rs62192851. The effect allele, with an allele frequency of 9.0% in both the FinnDiane and the DIREVA cohorts, was associated with a lower bacterial infection frequency, as opposed to the hypothesized increased susceptibility to infections. In the FinnDiane cohort, stratification according to the genotype of rs62192851 demonstrated markedly lower antibiotic purchase rates dose-dependently with increasing numbers of effect alleles (Fig. 3). Compared to homozygotic carriers of the reference alleles (N = 4231), homozygotic carriers of the effect alleles (N = 44) demonstrated a 37% lower antibiotic purchase frequency (0.60 [IQR 0.30–1.20] vs 0.38 [0.22–0.90] median of the average annual antibiotic purchase rate per subject, P = 0.01), while heterozygotic carriers of one effect allele (N = 817) had an 8% lower median purchase rate per subject (0.55 [0.29–1.05], P = 0.01) as compared to the homozygotic carriers of the reference allele. The difference in the antibiotic purchase frequencies between heterozygotic carriers and the homozygotic carriers of the effect allele was borderline-significant (P = 0.07).

### Replication analysis

We tested replication of all variants with P < 1 × 10^–5^ in the Swedish ANDIS cohort (All New Diabetics In Scania) of subjects with either type 1 or type 2 diabetes and in the FinnGen cohort of non-diabetic individuals. The top SNP, rs62192851, discovered in the meta-analyses was not associated with the phenotype in the ANDIS (rs62192851 P = 0.60) or the FinnGen-cohort (P = 0.52, Table 3). However, in the FinnGen-cohort, two variants (rs6727834 and rs10188087) from the lead locus were nominally replicated (P = 0.03 and P = 0.04, respectively).

**Table 3:** Replication of the top loci (P < 1 × 10–5) in the ANDIS (individuals with type 1 and type 2 diabetes) and the FinnGen (individuals without diabetes) replication cohorts. Table shows the top variant from the GWAS meta-analysis in each locus, as well as two additional variants that replicated in the FinnGen-cohort (rs6727834 and rs10188087 in the chromosome 2 locus).

SNP	Chromosome:position	EA/OA	Meta-analysis			ANDIS		FINNGEN	
			EAF	β (95%CI)	P	EAF	P	EAF	P
rs62192851	2:208,980,851	A/G	0.09	− 0.13 (− 0.18 to − 0.08)	2.23 × 10^–7^	0.09	0.60	0.08	0.52
rs6727834	2:208,960,779	T/G	0.07	− 0.15 (− 0.21 to − 0.09)	1.52 × 10^–6^	0.08	0.99	0.06	0.03
rs10188087	2:208,964,297	T/G	0.07	− 0.14 (− 0.20 to − 0.08)	3.31 × 10^–6^	0.08	0.95	0.06	0.04
rs35451602	2:3,631,189	T/TAA	0.20	− 0.09 (− 0.12 to − 0.05)	7.03 × 10^–6^	NA	NA	NA	NA
rs36002025	3:195,278,355	T/C	0.43	− 0.08 (− 0.11 to − 0.05)	1.30 × 10^–6^	0.42	0.98	0.46	0.40
rs4099475	4:37,773,400	C/G	0.05	− 0.16 (− 0.23 to − 0.09)	6.79 × 10^–6^	0.07	0.13	0.06	0.50
rs141002783	5:143,269,636	C/G	0.02	0.27 (0.15 to 0.39)	9.09 × 10^–6^	NA	NA	0.03	0.08
rs4559039	5:160,233,534	A/C	0.03	− 0.26 (− 0.36 to − 0.16)	3.08 × 10^–7^	0.05	0.93	0.03	0.83
rs183041036	7:158,289,622	G/A	0.22	0.11 (0.06 to 0.15)	4.03 × 10^–6^	NA	NA	NA	NA
rs7833059	8:70,952,739	C/G	0.09	− 0.12 (− 0.17 to − 0.07)	3.06 × 10^–6^	0.15	0.76	0.09	0.35
rs11110058	12:100,210,357	A/G	0.19	− 0.09 (− 0.13 to − 0.05)	7.30 × 10^–6^	0.20	0.15	0.18	0.09
rs7316104	12:93,918,909	C/T	0.44	− 0.07 (− 0.1 to − 0.04)	2.05 × 10^–6^	0.42	0.19	0.44	0.41
rs11850643	14:100,193,684	A/G	0.03	− 0.27 (− 0.38 to − 0.16)	1.26 × 10^–6^	0.03	0.48	0.03	0.74
rs2888050	14:81,588,015	T/G	0.20	0.08 (0.05 to 0.12)	5.65 × 10^–6^	0.21	0.77	0.20	0.56
rs1478709	16:7,221,984	A/G	0.08	0.12 (0.06 to 0.17)	7.92 × 10^–6^	0.87	0.40	0.08	0.41

*SNP* single nucleotide polymorphism, *EA/OA* effect allele/other allele, *EAF* effect allele frequency, *NA* not applicable.

### Exome sequencing analysis

We sought for common and rare coding (exonic) variants within the 500 kb region flanking the top 13 loci using whole exome sequencing (WES) data in the FinnDiane cohort (N = 368) and tested their association with infection susceptibility. Gene burden tests revealed two genes to be associated with the phenotype when restricted to missense mutation variants with any frequency: *CRYGB* (Crystallin Gamma B; P = 0.01) and *RELL1* (RELT-like protein 1; P = 0.03). *CRYGB* is located on chromosome 2 close to the lead locus of the present study. *RELL1* has been found to induce apoptosis of human epithelial cells through the activation of p38 MAPK pathway^23^. However, both *CRYGB* as well as *RELL1* remained as suggestive findings after adjustment for multiple testing (P_thresh_ = 0.0002, corrected for 208 genes within the 500 kb flanking region). No significant loss-of-function variants were found to associate with the phenotype. As the lead locus is within a non-coding region the WES data did not capture these variants.

### In silico analysis

#### Expression quantitative trait loci (eQTL)

The lead variant, rs62192851, as well as 18 other suggestive variants from the lead locus had been shown to affect the expression of *CRYGD* (Crystallin Gamma D) in heart tissue (Supplementary Table S1). *CRYGD* codes for crystallin proteins that make up the vertebrate lens, but how *CRYGD* could affect host defense mechanisms against pathogens is unclear.

#### Chromatin conformation capture data

Of the 21 SNPs from the lead locus with suggestive P-values, nine variants were on DNA fragments that interact with promoter regions of genes in open chromatin conformation capture data (Supplementary Table S1). Six of these variants were seen to bind to the promoter region of *DYTN* (Dystrophin) in immunologic cell lines and three interacted with the promoter regions of *CCNYL1* (Cyclin-Y-Like Protein 1). The exact role of *DYTN* is uncertain, but the gene has previously been associated with nephrolithiasis and encephalitis. *CCNYL1* has been associated with spermatogenesis but how the gene might affect infection susceptibility is unknown^24^.

#### Pathway analysis

We performed pathway analysis with the Pascal software to identify biological pathways enriched for association signal for infection susceptibility. Although no pathways were significant after correction for multiple testing, the most significant signaling pathway showed suggestive association and proved to be highly involved in immunological reactions: *IRAK1 recruit IKK complex* (P = 5.9 × 10^–4^). *IRAK1* (Interleukin 1 Receptor Associated Kinase 1) has been found to play a major role in initiating the innate immune response to microbial pathogens. Stimulation of *IRAK1* activates the Interleukin-1R/Toll-like receptor signaling pathway through interaction with Tumor Necrosis Factor receptor-associated factor 6 (TRAF6) resulting further downstream in the activation of the NF-κB, which finally leads to the initiation of immune and inflammatory responses^25^. Importantly, the *IRAK1* signaling pathway is triggered by microbial pathogens and structures, including bacterial lipopolysaccharides (LPS), components of the cell membrane in gram-negative bacteria.

## Discussion

In the present study, we hypothesized that genetic factors may affect the susceptibility to bacterial infections in individuals with diabetes. We performed a GWAS in two Finnish cohorts using comprehensive register data on prescription purchases of oral, systemic antibiotics, and corrected the analysis for glycated hemoglobin (HbA_1c_) levels, a known environmental risk factor for bacterial infections. In the meta-analysis combining the results from both GWAS cohorts, we discovered a locus on chromosome 2 with altogether 21 common variants reaching suggestive P-values and in high LD with one another. The top variant rs62192851 had a P-value of 2.23 × 10^–7^ in the meta-analysis. Moreover, stratification in the FinnDiane cohort according to the genotypes of the top variant showed that homozygotic carriers of the effect allele had a 37% lower median annual antibiotic purchase rate, as compared to homozygotic carriers of the reference alleles.

Although the association signal is located in a non-coding region, eQTL-data showed an association with the Crystallin Gamma protein family as nearly all variants in the lead locus were seen to affect the expression of *CRYGD*. The role of the crystallin gamma protein family in immunological reactions or pathways is uncertain, as it has previously mainly been attributed to protein structures in the lens. In open chromatin conformation capture data, the variants were also seen to interact with the promotor regions of *DYTN* (Dystrophin) and *CCNYL1B* (Cyclin Y Like 1). Fine-mapping of all the suggestive loci with gene burden tests using WES analysis revealed *CRYGB* and *RELL1* to be potentially associated with the phenotype in individuals with type 1 diabetes. Genome-wide pathway analysis using the P-values of the variants from the meta-analysis indicated that *IRAK1* and its signalling pathway that upregulates inflammatory responses upon stimulation by microbial pathogens through the NF-κB activation by IKK recruitment following IL1 and TLR activation, would be a mediator of our phenotype.

As we used a GWAS approach in our analysis we were unable to pinpoint the association of our phenotype with specific genes, although, in silico as well as WES analyses were performed to further study these associations. It is noteworthy, that two additional genes of interest were within 200 kB of our lead locus, previously associated with immunological reactions and host defense mechanisms against pathogens: *IDH1* and *PIKFYVE. IDH1* is known to participate in epigenetic remodeling in cells with myeloid lineage^26^, while *PIKFYVE* (Phosphatidylinositol-3-phosphate 5-kinase) in turn has been found to affect the replication of viruses and intracellular bacteria^27,28^. Interestingly, this gene has further been found to affect GLUT4 translocation and has therefore been linked to insulin-dependent glucose transport^29^.

We found no significant associations with our phenotype in the replication analysis in the Swedish ANDIS cohort, which might suggest that our finding is unique for the Finnish population. This notion is also supported by the fact that two variants from the lead locus replicated nominally in the Finnish FinnGen cohort in individuals without diabetes. This potentially further demonstrates that the findings are not necessarily diabetes-specific but applicable to non-diabetic individuals as well. However, the possibility of a false positive finding in the present study must be acknowledged, as no variants attained GWAS-significance in the meta-analysis and only two variants from the lead locus marginally replicated in the FinnGen-cohort. Therefore, our results must be interpreted with caution, until validation studies are conducted.

Our GWAS is unique in that we relied on national register data from Finland and Sweden on antibiotic prescription purchases instead of recorded diagnoses of bacterial infections. This was feasible as the National Drug Prescription Purchase Register in Finland reliably reflects the bacterial infections treated in outpatient care, as antibiotics in Finland may not be purchased without a prescription. This method also allowed us to include large population cohorts, as register data on prescription purchases are available for research purposes and offer extensive retrospective data. Furthermore, the use of data on antibiotic purchases made it possible to investigate the general susceptibility by including all types of antibiotics, reflecting every bacterial infection treated in outpatient care.

We also note some shortcomings in our study. As the phenotype only includes outpatient prescriptions, severe infections treated in hospitals were not included. Furthermore, we have previously shown that the presence and severity of diabetic kidney disease is a strong risk factor for bacterial infections. As we did not adjust our GWAS-analysis for stage of diabetic kidney disease or kidney function, we cannot exclude the possibility that our findings are associated with diabetic kidney disease rather than with bacterial infections. However, in the FinnDiane cohort we saw how the allele carrier status of our top variant rs62192851 significantly affected the antibiotic purchase frequency, which supports the association between this locus and bacterial infection frequency. We also censored the follow-up at the onset of end-stage kidney disease, and further adjusted our analysis for glycemic control. As glycemic control strongly correlates with the severity of diabetic kidney disease, this indirectly adjusted the analysis for diabetic kidney disease as well, and further suggests that our findings and results associate to bacterial infections. On the other hand, it is possible that the genetic factors predisposing individuals with diabetes to infections act through hyperglycemia associated pathways and due to the adjustment for glycemic control, these potential findings were not investigated in the present study.

Clinicians may have a lower threshold for prescribing antibiotics to individuals with diabetes. This could affect how well our results can be extrapolated to the healthy non-diabetic population. Our data also included prophylactic antibiotic purchases, however, we argue that this still reflects an increased risk for an infection that required antibiotic treatment, which, in turn reflects the susceptibility to infections. As all subtypes of oral antibiotics were included in the study, our phenotype can be considered quite heterogenous. This may have diluted the significance and affected the statistical power, as opposed to studying specific infections, which could have resulted in a more specific phenotype. However, due to the heterogeneity, this could also potentially imply that our findings may demonstrate biological common immunity checkpoints for immunologic reactions concerning several different bacterial pathogens.

To conclude: the present study is to our knowledge the first to explore the potential genetic factors affecting infection susceptibility in individuals with diabetes. Our results indicate that common variants on chromosome 2, captured by GWAS analysis, may be associated with a reduced risk of infections in individuals with diabetes. Pathway-analysis suggested that genetic variation in the IRAK1-pathway is also involved in this infection susceptibility. More studies are needed to further elucidate the genetic factors behind the increased susceptibility to bacterial infections in individuals with diabetes.

## Methods

### Cohorts and data collection

**Figure 4:**
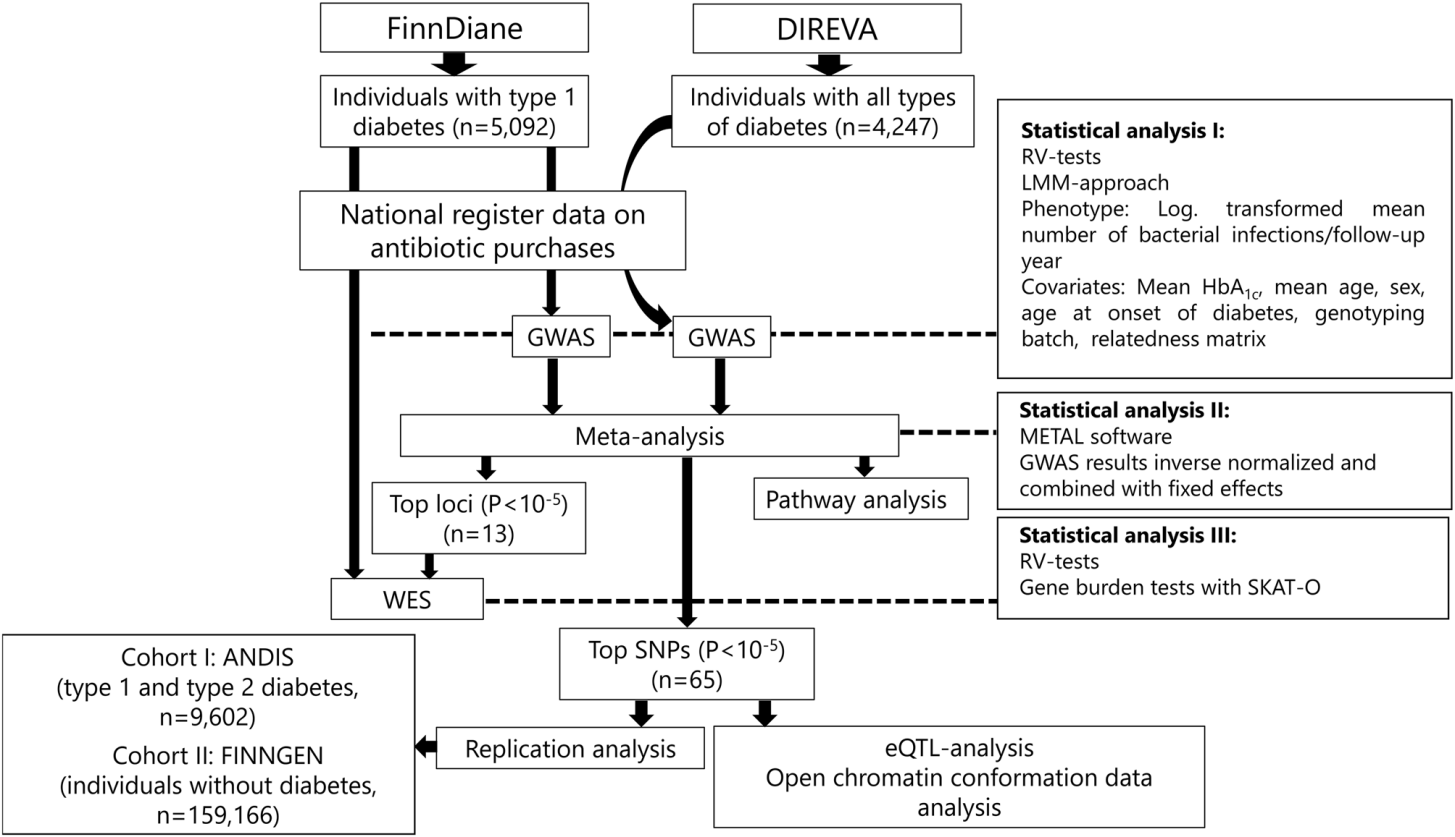
Flow chart summarizing the study design. *FinnDiane* Finnish Diabetic Nephropathy study, *DIREVA* Vaasa Diabetes Registry, *ANDIS* All New Diabetics In Scania, *GWAS* Genome Wide Association Study, *SNP* Single Nucleotide Polymorphism, *WES* Whole Exome Sequencing, *eQTL* Expression quantitative trait loci, *LMM-approach* linear mixed model approach, *HbA*_*1c*_ glycated haemoglobin, *SKAT-O* Sequence Kernel Association Optimal Unified Test.

For the main analyses, individuals were included from two separate Finnish cohorts: individuals with type 1 diabetes (N = 5092) from the FinnDiane study, and individuals with all types of diabetes (N = 4247) from the DIREVA study (Fig. 4). FinnDiane is an ongoing nationwide multicenter survey, founded in 1997 to elucidate genetic and environmental risk factors for diabetic complications with an emphasis on diabetic nephropathy^30^. DIREVA is an ongoing regional study, founded in 2007 to improve and personalize the treatment of diabetes and includes over 7000 individuals with diabetes from the Vaasa hospital district. In the FinnDiane cohort, type 1 diabetes was defined as onset of diabetes before 40 years of age and start of insulin treatment within one year from the onset. The FinnDiane protocol is in accordance with the Declaration of Helsinki and has been approved by the local ethics committees at each study center. The Direva Study has been approved by the ethics committees of Vaasa Hospital District and Turku University Hospital. Prior to the participation, all participants gave their written informed consent.

Bacterial infections were identified using comprehensive nationwide register data. Data on antibiotic prescription purchases were collected between the 1st of January 1995 to the 31st of December 2014 from the Finnish National Drug Prescription Register. All systemic oral antibiotics were identified from the register data, using the Anatomical Therapeutic Chemical (ATC) Classification systems code J01. In Finland, oral, systemic antibiotics are not available over the counter in pharmacies and require a prescription from a physician. Consequently, antibiotic purchases listed in the prescription purchase register reflect the diagnoses of bacterial infections treated in outpatient care.

### Genotyping, imputation and statistical analysis

DNA samples were genotyped using the HumanCoreExome BeadChips-12 v. 1.0, -12 v. 1.1, or -24 v. 1.0 BeadChip (Illumina, San Diego, CA) in both cohorts. After quality control filters (minor allele frequencies [MAF] ≥ 0.01 and imputation info r^2^ ≥ 0.7), genotype imputation with minimac 3 software^31^ and 1000 Genomes reference panel resulted in 8.4 × 10^6^ and 8.6 × 10^6^ SNPs in FinnDiane and DIREVA, respectively. In the GWAS analyses, estimated allele dosages were used and the analyses were performed with the RVTESTS software^32^, using a linear mixed model. To assess infection frequencies, an infection susceptibility risk score was calculated for all subjects as the logarithmically transformed mean number of antibiotic purchases per follow-up year. Due to an excess of zeroes in the data, a small constant (0.5 × minimum non-zero value) was added to the data before log_e_ transformation. As end-stage kidney disease increases the risk of infections dramatically, follow-up years during or after which individuals were diagnosed with end-stage kidney disease were censored. Follow-up years prior to the onset of diabetes were also excluded. As poor glycemic control is a well-known environmental risk factor for infections, the mean HbA_1c_ for each subject during the follow-up was calculated and added as a covariate in the analysis. To summarize, the following covariates were included in the GWAS-analysis: average age during follow-up, sex, mean HbA_1c_ during follow-up, age at onset of diabetes as well as genotyping batch-components and the kinship matrix. Results from the GWAS analyses in the FinnDiane and the DIREVA cohorts were inverse normalized and combined with fixed effects meta-analysis using METAL software.

### Narrow sense heritability

The proportion of the phenotypic variance attributable to additive genetic factors was estimated in the FinnDiane cohort using a genetic relationship matrix (GRM) of unrelated individuals in a mixed linear model, via the restricted maximum likelihood (REML) approach. This was performed using the Genome-wide Complex Trait Analysis (GCTA) software^33^.

### Replication cohorts

SNPs with a P-value < 1 × 10^–5^ were included in the replication analyses performed in two separate cohorts: the Swedish ANDIS-cohort and the Finnish FinnGen-cohort. The ANDIS-cohort consisted of individuals with both type 1 and type 2 diabetes (n = 9602) and the FinnGen-cohort consisted of individuals without type 1 or type 2 diabetes and an age above 35 years (n = 159,166). Similar to the main cohorts, antibiotic purchases were identified using the ATC-code of J01. The phenotype in the FinnGen-cohort was defined as the total number of antibiotic purchases during the follow-up, with further adjustment for age, sex, genotyping batch and principal components. Details on the ANDIS and FinnGen studies have been reported previously^34,35^.

### Exome sequencing analysis

Whole exome sequencing (WES) data on individuals from the FinnDiane cohort (N = 368) was used to fine-map the exon-regions of the most significant loci (P < 1 × 10^–5^) discovered in the meta-analysis. From each locus the top variant was included in the analysis and all genes within a region of 0.5 Mb up- and downstream of these variants were identified. Gene burden tests for these genes were used for the identification of missense and loss-of-function-variants using the gene aggregate test SKAT-O. Gene burden tests were conducted with variant frequency filters of 0.05 and 0.5. WES analyses were performed with RVTESTS-software.

### In silico analysis

#### Pathway analysis

As pathway analysis improves statistical power and facilitates significant discoveries even in small sample sets with complex phenotypes, we performed pathway scoring with the Pascal software^36^. This software utilizes P-values of the variants from the GWAS summary statistics and uses a modified Fisher method, which eliminates the need for arbitrary significance-threshold selection and yields power improvement. We included variants with a MAF > 0.01 in the analysis and genes with less than 3000 variants. Significant P-value thresholds were calculated with correction for multiple testing: P_thresh_ = 2.9 × 10^–5^.

#### Chromatin conformation capture data

Variants in non-coding regions may affect the expression of genes not necessarily close in genetic proximity by physically interacting with the promoter regions of the genes due to chromatin conformation. To study these genetic interactions of our findings, we used the freely available 3D chromatin conformation capture data ChiCp (Promoter Capture Hi-C) on chicp.org^37^. A systematic search was performed on all suggestive variants (P < 1 × 10^–5^) by separately inserting them into the ChiCp-browser. The level of significance of the interaction score in the tests was set to a score over 5. Interactions were studied in macrophage M0-M2-cells, monocytes, neutrophils and finally Naïve as well as Total B-, CD4 and CD8 cells. Only the most significant finding for each variant is reported.

#### Expression quantitative trait loci (eQTL)

For all suggestive variants (P < 1 × 10^–5^) potential expression quantitative trait loci (eQTL) were further studied using the Genotype-Tissue Expression project portal (GTEx V7, https://www.gtexportal.org/home/). Only the most significant eQTL is reported for each variant.

## Supplementary Information

Supplementary Information.

## Data Availability

The ethics statement and the informed consent do not allow sharing of individual-level data.
