# Supplementary material for: Genetic factors affect the susceptibility to bacterial infections in diabetes
Source: Sci Rep. 2021 May 4;11:9464. doi: 10.1038/s41598-021-88273-w (PMC8096814; doi:10.1038/s41598-021-88273-w)
Supplement: Supplementary file 1 — Supplementary Information. [file 41598_2021_88273_MOESM1_ESM.pdf]

Supplementary Information

Genetic factors affect the susceptibility to bacterial infections in diabetes.

Johan R. Simonsen[1,2,3], Annemari Käräjämäki[4,5], Anni A. Antikainen[1,2,3], Iiro Toppila[1,2,3], Emma Ahlqvist[6], Rashmi Prasad[6], Dina M. Aly[6], Valma Harjutsalo[1,2,3,7], Asko Järvinen[8], Tiinamaija Tuomi[1,3,5,9,10], Leif Groop[6,10] , Carol Forsblom[1,2,3], Per-Henrik Groop[1,2,3,11], Niina Sandholm[1,2,3] and Markku Lehto[1,2,3]

[1]Folkhälsan Institute of Genetics, Folkhälsan Research Center, Helsinki, Finland;

[2]Abdominal Center, Nephrology, University of Helsinki and Helsinki University Hospital, Helsinki, Finland; [3]Research Program for Clinical and Molecular Metabolism, Faculty of Medicine, University of Helsinki, Finland;

[4]Department of Primary Health Care, Vaasa Central Hospital, Vaasa, Finland;

[5]Diabetes Center, Vaasa Health Care Center, Vaasa, Finland

[6]Department of Clinical Sciences, Lund, University Diabetes Centre, Lund University, Skåne University Hospital, Malmö, Sweden, Lund, Sweden;

[7]National Institute for Health and Welfare, Helsinki, Finland;

[8]Division of Infectious Diseases, Inflammation Centre, Department of Medicine, Helsinki University Hospital, Helsinki, Finland;

[9]Abdominal Center, Endocrinology, University of Helsinki and Helsinki University Hospital, Helsinki, Finland

[10]Institute for Molecular Medicine Finland, Helsinki, Finland

[11]Department of Diabetes, Central Clinical School, Monash University, Melbourne, Victoria, Australia.

Supplementary table S1. Summary of expression quantitative trait loci (eQTL) and interactions with promotor regions of genes in open chromatin conformation capture data of the top variants in the lead locus as well the top variant from each suggestive loci ($p<1\times10^{-5}$) discovered in the meta-analysis.

SNP	Chromosome:Position	P (meta-analysis)	eQTL-analysis			Open chromatin conformation capture data		
			eQTL Gene	Tissue	P	Gene	Cell Line	Score
rs6727834	2:208960779	3.31×10^{-6}	CRYGD	Heart - Atrial Appendage	6.3×10^{-17}	CCNYL1	Macrophages M0	7.01
rs10188087	2:208964297	3.10×10^{-6}	CRYGD	Heart - Atrial Appendage	8.0×10^{-17}	CCNYL1	Macrophages M0	7.01
rs6435409	2:208967087	1.15×10^{-6}	CRYGD	Heart - Atrial Appendage	1.0×10^{-16}	CCNYL1	Macrophages M0	7.01
rs6435410	2:208971883	1.26×10^{-6}	CRYGD	Heart - Atrial Appendage	2.1×10^{-17}	NA	NA	NA
rs10578621	2:208972554	1.14×10^{-6}	CRYGD	Heart - Atrial Appendage	2.1×10^{-17}	NA	NA	NA
rs12624138	2:208973011	7.30×10^{-6}	CRYGD	Heart - Atrial Appendage	2.1×10^{-17}	NA	NA	NA
rs6711600	2:208973323	1.17×10^{-6}	CRYGD	Heart - Atrial Appendage	2.1×10^{-17}	NA	NA	NA
rs6711708	2:208973387	1.14×10^{-6}	CRYGD	Heart - Atrial Appendage	2.1×10^{-17}	NA	NA	NA
rs6711724	2:208973451	1.15×10^{-6}	CRYGD	Heart - Atrial Appendage	2.1×10^{-17}	NA	NA	NA
rs6759064	2:208973961	1.22×10^{-6}	CRYGD	Heart - Atrial Appendage	2.1×10^{-17}	NA	NA	NA
rs6435412	2:208976408	1.97×10^{-6}	CRYGD	Heart - Atrial Appendage	2.1×10^{-17}	NA	NA	NA
rs11679982	2:208977514	2.23×10^{-6}	NA	NA	NA	NA	NA	NA
rs62192824	2:208977960	2.13×10^{-6}	CRYGD	Heart - Atrial Appendage	1.6×10^{-15}	NA	NA	NA
rs58912287	2:208979179	2.23×10^{-7}	NA	NA	NA	NA	NA	NA
rs62192851	2:208980851	1.30×10^{-6}	CRYGD	Heart - Atrial Appendage	1.6×10^{-15}	NA	NA	NA
rs10176291	2:208982076	1.58×10^{-6}	CRYGD	Heart - Atrial Appendage	2.2×10^{-15}	DYTN	Total CD4 MF	8.33
rs145459319	2:208983357	5.93×10^{-6}	CRYGD	Heart - Atrial Appendage	1.6×10^{-15}	DYTN	Total CD4 MF	8.33
rs6435414	2:208983538	1.67×10^{-6}	CRYGEP	Brain - Hypothalamus	1.1×10^{-13}	DYTN	Total CD4 MF	8.33
rs62192852	2:208984202	3.05×10^{-6}	CRYGD	Heart - Atrial Appendage	1.6×10^{-15}	DYTN	Total CD4 MF	8.33
rs966932	2:208985903	3.47×10^{-6}	CRYGEP	Brain - Hypothalamus	4.7×10^{-14}	DYTN	Total CD4 MF	8.33
rs6435415	2:208986049	7.03×10^{-6}	CRYGEP	Brain - Hypothalamus	1.1×10^{-13}	DYTN	Total CD4 MF	8.33

rs35451602	2:3631189	2.48×10^{-6}	COLEC11	Skin - Not Sun Exposed (Suprapubic)	3.7×10^{-29}	ADI1	Total B	26.73
rs36002025	3:195278355	7.12×10^{-6}	AC091633.3	Testis	9.1×10^{-61}	LSG1	Naive CD4	8.71
rs4099475	4:37773400	7.85×10^{-6}	NA	NA	NA	RELL1	Total CD4 NonActivated	6.79
rs141002783	5:143269636	3.08×10^{-7}	NA	NA	NA	NA	NA	NA
rs4559039	5:160233534	6.04×10^{-7}	NA	NA	NA	NA	NA	NA
rs183041036	7:158289622	3.06×10^{-6}	NA	NA	NA	NA	NA	NA
rs7833059	8:70952739	3.30×10^{-6}	NA	NA	NA	NA	NA	NA
rs11110058	12:100210357	9.70×10^{-6}	NA	NA	NA	NA	NA	NA
rs7316104	12:93918909	6.77×10^{-6}	UBE2N	Skin - Sun Exposed (Lower leg)	7.8×10^{-10}	CRADD	Monocytes	5.29
rs11850643	14:100193684	8.71×10^{-6}	DEGS2	Cells - Cultured Fibroblasts	6.6×10^{-5}	EML1	Naive CD4	13.74
rs2888050	14:81588015	6.05×10^{-6}	NA	NA	NA	GTF2A1	Monocytes	9.37
rs1382484	16:7218129	7.92×10^{-6}	NA	NA	NA	NA	NA	NA

SNP indicates Single Nucleotide Polymorphism; eQTL, expression quantitative trait loci; NA, not applicable

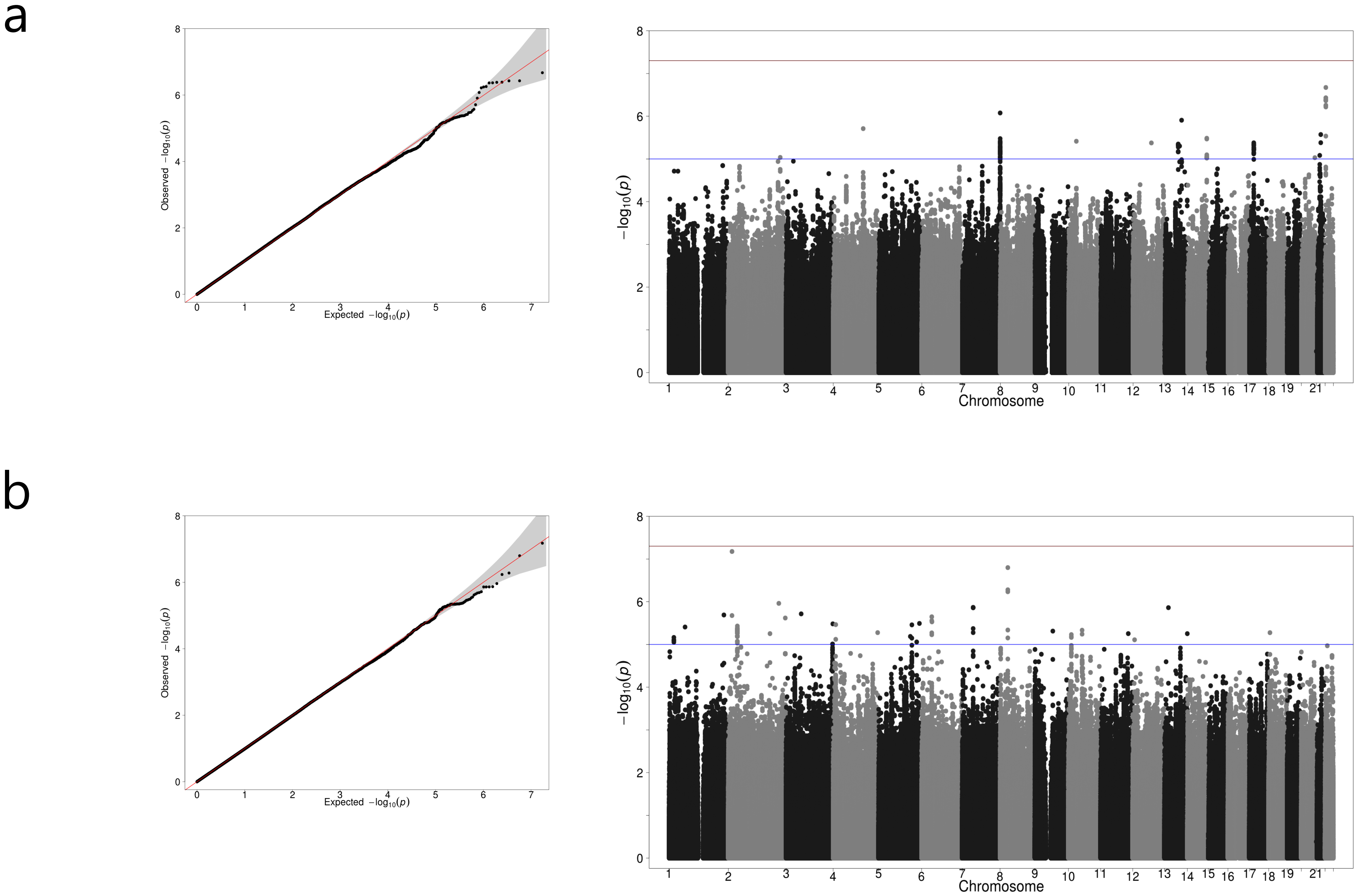

Supplementary Figure S1. Manhattan- and quantile-quantile plots of the GWAS in the a) FinnDiane-cohort and b) DIREVA-cohort. Two horizontal lines indicate P value thresholds for suggestive significance ($P<10^{-5}$) and genome-wide significance ($<5\times10^{-8}$).
